# The Baltic CAI challenge: reconciling Transatlanticism with EU solidarity

**DOI:** 10.1007/s10308-021-00625-3

**Published:** 2021-06-09

**Authors:** Una Aleksandra Bērziņa-Čerenkova

**Affiliations:** 1 Riga Stradins University, Riga, Latvia; 2 Latvian Institute of International Affairs, Riga, Latvia

## Abstract

As the EU officials and their Chinese counterparts emphasised the end of 2020 as the date for a successful conclusion of the Comprehensive Agreement on Investment (CAI, the Agreement), the Baltic states of Estonia, Latvia, and Lithuania were sceptical. However, after discussions, with Lithuania appearing to be the most visible opponent of CAI among the Baltic nations, all three eventually upheld the proposal. Understanding that the ratification of CAI is unlikely after the mutual exchange of sanctions between the EU and PRC in March, 2021, the report nevertheless examines the roots of the Baltic position as a case study of inter-EU bargains, inspects what factors contributed to the Baltic position on the issue of CAI, presents the national pro- and counter-arguments to CAI along the domains of geo-politics, values, and economy, and brings up the dilemmas that remain unsolved.

## The Transatlantic argument

The Transatlantic link is at the cornerstone of the three Baltic states’ foreign and security policies. With the renewed possibility of the Russian threat to the sovereignty of Lithuania, Latvia, and Estonia, especially post-2014, the Baltic three are not just NATO members, but strong proponents of bilateral ties with the USA, fostering policy commitments based on personal relationships, and lobbying for the national interests in Washington. This approach is historically justified—after all, the key document stating the United States’ non-recognition policy of Soviet annexation^1^ of 1940 at the initiative of the acting Secretary of State Sumner Welles, which “established an international framework for the survival of the three Baltic states de jure throughout the entire period of Soviet occupation and for the eventual restoration of statehood of Estonia, Latvia and Lithuania”,^2^ was authored by Loy W. Henderson, a US diplomat with strong personal and professional links to Latvia.^3^

The Baltics constantly reaffirm the importance of the relationship with the USA and argue in favour of closer EU-US cooperation. Estonia names “reinforcing the transatlantic link both in the framework of NATO as well as in European Union–United States cooperation” an integral part of a strengthening Europe.^4^ Lithuania defines “strengthening the EU-US cooperation” as one of its foreign policy objectives.^5^ Similarly, Latvia positions itself as “a country that strengthens Europe and transatlantic links with the United States and Canada”,^6^ and supports the US position on the majority of security-related issues.

Therefore, all three countries initially were weary of the hurriedness of the process and the lack of coordination with the USA, especially since the EU willingness to jointly negotiate its position on China could have served as a natural restart of EU-US relations under the new Biden administration.

As Jake Sullivan, Joe Biden’s National Security Adviser, called for more EU-US coordination on “concerns about China’s economic practices”,^7^ implicitly condemning the EU’s rush to sign the CAI in the eleventh hour, the Baltics were conflicted—the historical context and the haste made CAI “a Transatlantic problem first and foremost”^8^ for Tallinn, Riga, and Vilnius alike. Even though challenging European unity on this particular issue proved to be too “high-stakes” for the three countries, the dilemma once again revealed the Baltic sentiment against an overly self-reliant European autonomy outlook.

## CAI pros and cons: the national rationale

As elsewhere in Europe, the debate over the Baltic national positions on CAI can be grouped into three topics: geopolitical implications, values, and economy. Since the former has already been addressed in the previous sub-chapter, the following section provides an examination of the two latter considerations.

From the value perspective, the most visible argument against CAI stems from the fear of becoming effectively complicit to forced labour practices exercised by China. Under the current non-finalised edition, CAI Section IV states that “the Parties agree to promote investment policies which further the objectives of the Decent Work Agenda.. including a human-centred approach to the future of work, adequate minimum wages, social protection and safety and health at work.”^9^ And yet, at the level of the national parliaments, questions arise on whether, first of all, signing CAI while knowing of such practices is ethical, and, secondly, if the Agreement would be an effective instrument to ensure accountability.^10^ Although the forced labour issue is wider than a given ethnic group, still, the debate became particularly electrified as information appeared that China’s exports to the EU, e.g., solar panel components,^11^ could have been produced by the detained members of the Uighur minority. Lithuania, which has become a particularly value discourse-driven player vis-a-vis China since 2019, ultimately did decide in favour of going along with CAI, but demonstrated its position as the parliamentary committees on foreign affairs and national security decided to draft a resolution on the persecution of Uighurs in China less than two months after the inking of the Agreement.^12^ Admittedly, the Lithuanian agenda regarding CAI was wider than just grievances over values—the country saw the debate over CAI as an opportunity to reiterate its fears over the nuclear power plant situated just 20 km from the Lithuanian border in Astravyets, Belarus, by calling on the European Commission to include a clause on banning electricity imports from the plant^13^—even though the claim did not materialise, it certainly succeeded at attracting attention to the issue.

The debate over the economic benefits boils down to the question: what do the Baltics have to gain from CAI given the humble track record of investment and export so far and the shrinking room for cooperation in light of US-China disputes and security concerns? The Baltic states are not avid investors in China, particularly not in the sectors the EU companies are players in (automotive, basic materials, finance account for 59% of EU investments in China^14^). As colourfully worded by Chairman of the Board and Chief Executive Officer of the microwave data transfer solutions producer SAF Tehnika N.Bergs, “We have been working with China for around twenty years in different forms. My first reaction to this document [CAI] is—we don’t know hell. We don’t know anything. We can speculate. Secondly, I don’t know any Latvian companies that have significant investment in China.”^15^ Indeed, Latvian investors have no major projects in China—the scale is within several tens of thousands of EUR.^16^

Nevertheless, whilst severely Trans-Atlanticist in their defence thinking, as outlined above, the Baltic states do understand the benefits of belonging to a larger grouping when it comes to economic relations—any deal that gives more investment opportunities while offering protection, and aiming at reciprocity, at least on paper, is better than no deal at all.^17^ The EU as a joint bloc possesses the kind of leverage that individual countries just don’t have.

Also, the Agreement would allow the EU as a bloc to equalise its economic relations with China, and the Baltic states benefit by extension if more balance is achieved. Finally, the Agreement could be useful for Baltic companies after all: even if they are not necessarily investors in China themselves, they are part of larger European value chains who very well are.^18^ These points resonate with the local business communities and larger populations. Research in Latvia (other Baltic states were not included) shows that the public still upholds the economic opportunities narrative—over 80% of respondents believe investment and trade promotion should be the utmost priority for the national foreign policy towards China.^19^

But it is also possible that any gains will be further limited by China. MEP Reinhard Butikofer (Alliance 90/The Greens) has reported on some backchannel signals from the Chinese side that, if true, are particularly alarming to the Baltic states: “I can’t prove this, but I hear that in the so-called ‘legal scrubbing’ phase of work on CAI the Chinese side is trying to re-introduce their request that member states which limit Huawei access to 5G networks should not benefit from market opening in China.”^20^ Estonia, Latvia, and Lithuania have all signed Joint Declarations on 5G with the USA, declaring the “desire to strengthen our cooperation on 5G”^21^ and putting forward a list of requirements on financing, government control, IPR, et al., aimed specifically at limiting China. Although it is clear that no position that excludes a number of member states could ever be supported by the EU, still, if China is indeed thinking along the lines of curbing the especially US-sympathetic and NATO-minded EU countries, the Baltics could find themselves at the receiving end of subtle and undetectable instruments of exclusion, rendering any gains from EU-China agreements ultimately inaccessible to them.

If in 2013 the EU feared that the European members of the “16 + 1” platform, including the Baltic states, could become pawns in Beijing’s play at dividing the EU^22^—then today in the aftermath of CAI some analysts in Beijing speak of the Baltic states defying EU unity again—only this time by roaming too close to the categorical US position.^23^ Thus, ironically, the region’s China policies are being singled out as a risk to EU unity once more, but in an absolutely contrary sense—from being too China-optimistic to being too US-reliant.

Ultimately, the CAI ratification is unlikely after the March 2021 exchange of sanctions, but the analysis of the Baltic perceptions and the internal and EU-wide bargaining serves as a valuable case study of EU differences in expectations and interests towards China, as well as the strong role the Transatlantic relationship plays in the Baltic China positions.

## Footnotes

1 “Welles Declaration, Department of State Press Release, ‘Statement by the Acting Secretary of State, the Honorable Sumner Welles’”, July 23, 1940, History and Public Policy Program Digital Archive, NARA, RG 59, https://digitalarchive.wilsoncenter.org/document/144967

2 “Joint Statement by the U.S. Secretary of State and the Ministers of Foreign Affairs of Estonia, Latvia, and Lithuania”, US Embassy in Latvia, July 22, 2020, https://lv.usembassy.gov/joint-statement-by-the-u-s-secretary-of-state-and-the-ministers-of-foreign-affairs-of-estonia-latvia-lithuania/

3 Sils, Kārlis, “Welles Declaration: Personal tragedy led to 50 years of protecting Baltic statehood,” Latvian Public Broadcasting, March 20, 2019, https://eng.lsm.lv/article/culture/history/welles-declaration-personal-tragedy-led-to-50-years-of-protecting-baltic-statehood.a313352/

4 “Report on foreign policy by Minister of Foreign Affairs Eva-Maria Liimets at the Parliament of Estonia,” Republic of Estonia, Ministry of Foreign Affairs, February 16, 2021, https://vm.ee/en/news/report-foreign-policy-minister-foreign-affairs-eva-maria-liimets-parliament-estonia

5 “Resolution of the Seimas of the Republic of Lithuania on Directions in Foreign Policy,” Ministry of Foreign Affairs of the Republic of Lithuania, December 10, 2020, https://urm.lt/default/en/foreign-policy/key-foreign-policy-documents/resolution-of-the-seimas-of-the-republic-of-lithuania-on-directions-in-foreign-policy-may-1-2004

6 “Speech by Foreign Minister Edgars Rinkēvičs at the annual Foreign Policy Debate in the Latvian Parliament (Saeima), 28 January 2021,” Ministry of Foreign Affairs, Republic of Latvia, January 28, 2021, https://www.mfa.gov.lv/en/news/latest-news/67403-speech-by-foreign-minister-edgars-rinkevics-at-the-annual-foreign-policy-debate-in-the-latvian-parliament-saeima-28-january-2021

7 Jake Sullivan (@jakesullivan), Twitter thread, December 22, 2020, https://twitter.com/jakejsullivan/status/1341180109118726144

8 Kuusik, Piret, “EU-China Investment Deal: A Transatlantic Problem First and Foremost,” International Centre for Defence and Security, January 15, 2021, https://icds.ee/en/eu-china-investment-deal-a-transatlantic-problem-first-and-foremost/

9 “Section IV: Investment and sustainable development, Sub-Sect. 3, Article 5,” January 22, 2021, https://trade.ec.europa.eu/doclib/docs/2021/january/tradoc_159346.pdf

10 The European Affairs Committee of the Saeima, Meeting regarding the Approval of the National position on CAI, February 26, 2021.

11 Hernández-Morales, Aitor, et.al. “Fears over China’s Muslim forced labor loom over EU solar power,” Politico, February 10, 2021, https://www.politico.eu/article/xinjiang-china-polysilicon-solar-energy-europe/

12 “Lithuanian parliament drafts resolution on Uighur persecution in China,” LRT, Source: BNS, February 17, 2021, https://www.lrt.lt/en/news-in-english/19/1346441/lithuanian-parliament-drafts-resolution-on-uighur-persecution-in-china

13 “Lithuania calls for Belarus nuclear energy ban in EU–China pact,” LRT, source: BNS, December 30, 2020, https://www.lrt.lt/en/news-in-english/19/1309490/lithuania-calls-for-belarus-nuclear-energy-ban-in-eu-china-pact

14 “EU AND CHINA COMPREHENSIVE AGREEMENT ON INVESTMENT factsheet,” European External Action Service, https://eeas.europa.eu/sites/eeas/files/cai_fact_sheet_en.pdf

15 Bergs Normunds, interview in “ES un Ķīnas investīciju līgums | #Krustpunktā,” 15:46–16:32, LSM, January 28, 2021, https://www.facebook.com/LatvijasRadio1/videos/3641807542600472

16 Report by the Latvian Investment and Development Agency Economic Counsellor in Southern China Agnese Stūrmane to the European Affairs Committee of the Saeima, February 26, 2021.

17 Author’s Interview with Prof. Inna Šteinbuka, Chairwoman of the Fiscal Discipline Council of Latvia, February 9, 2021.

18 Presentation of the Latvian National position from: Report by Parliamentary Secretary of the Latvian Ministry of Foreign Affairs Ms. Zanda Kalniņa-Lukaševica to the European Affairs Committee of the Saeima, February 26, 2021.

19 Bērziņa-Čerenkova, Una, Turcsányi, Richard, Šimalčík, Matej, Kironská, Kristina, Sedláková, Renata, “Latvian public opinion on China in the age of COVID-19 Economic optimism against all odds,” Central European Institute of Asian Studies, 2021.

20 Reinhard Bütikofer (@bueti), Twitter thread, January 25, 2021, https://twitter.com/bueti/status/1353755391763607557

21 “United States–Estonia Joint Declaration on 5G Security,” U.S. Embassy in Estonia, November 1, 2019, https://ee.usembassy.gov/joint-declaration-on-5g/

22 E.g. Brenner, Thorsten et.al., “Authoritarian Advance: Responding to China’s Growing Political Influence in Europe,” Global Public Policy Institute, February 5, 2018, https://www.gppi.net/2018/02/05/authoritarian-advance-responding-to-chinas-growing-political-influence-in-europe

23 Private interview, anonymised by Author at the Interviewee’s request, January 11, 2021.

